# Longitudinal effects of choir singing on aging cognition and wellbeing: a two-year follow-up study

**DOI:** 10.3389/fnhum.2023.1174574

**Published:** 2023-07-20

**Authors:** Emmi Pentikäinen, Lilli Kimppa, Anni Pitkäniemi, Outi Lahti, Teppo Särkämö

**Affiliations:** ^1^Cognitive Brain Research Unit, Department of Psychology and Logopedics, Faculty of Medicine, University of Helsinki, Helsinki, Finland; ^2^Centre of Excellence in Music, Mind, Body, and Brain, University of Helsinki, Helsinki, Finland; ^3^Seinäjoki Central Hospital, Geriatric Outpatient Clinic, Rehabilitation Analysis Clinic, Seinäjoki, Finland

**Keywords:** choir singing, aging, quality of life, cognition, cognitive reserve

## Abstract

**Introduction:**

While increasing evidence points toward the benefits of musical activities in promoting cognitive and emotional well-being in older adults, more longitudinal studies are needed to establish their long-term effects and uncover the mechanisms through which musical activities affect well-being. Most previous research has focused on instrumental musical activities, but little is currently known about the long-term effects of singing, even though neuroimaging evidence suggests that it is a versatile activity for the brain, involving a multitude of neural processes that are potentially beneficial for well-being.

**Methods:**

We conducted a 2-year follow-up study to assess aging-related changes in cognitive functioning and emotional and social well-being with self-report questionnaires and standardized tests in 107 older adult choir singers and 62 demographically matched non-singers. Data were collected at baseline (T1), and at 1-year (T2) and 2-year (T3) follow-ups using questionnaires on subjective cognitive functioning, depression, social engagement, and quality of life (QOL) in all participants and neuropsychological tests in a subgroup of participants (45 choir singers and 41 non-singers).

**Results:**

The results of linear mixed model analysis showed that in verbal flexibility (phonemic fluency task), the choir singers had higher scores already at T1 and showed no change over time, whereas the non-singers showed enhancement from T1 to T3. Furthermore, active retrieval of word knowledge (WAIS-IV Vocabulary task) showed significantly different changes from T1 to T2 between the groups (enhancement in choir singers and decline in non-singers), however lacking significant change within groups. Similar opposite trajectories of QOL related to social inclusion and safety of the environment (WHOQOL-Bref Environmental subscale) were significant from T1 to T3, but these changes were not significant within groups or at each timepoint. Within the choir singers, shorter experience in choir singing was associated with greater improvement in the vocabulary task over the follow-up period, suggesting that initiation of choir singing at older age induces some verbal benefits. There were no group differences in any other questionnaire or neuropsychological measure over time.

**Discussion:**

In conclusion, our results suggest that choir singing at older age is associated with a sustained enhancement of phonemic fluency, while the effects on other verbal skills and quality of life are less clear.

## Introduction

Although the aging of the population reflects a multitude of advances made in health sciences, there is a discrepancy between the duration of life and the quality of life in older adults: while we now live longer, the number of healthy years has not increased accordingly (Garmany et al., 2021). Longer lifespans have increased, and keep increasing, the prevalence of age-related diseases, especially neurodegenerative diseases such as Alzheimer’s disease. Thus, there is a growing need for research on tools and interventions aimed at preventing aging-related diseases and promoting wellbeing in later life.

The natural aging process, driven by different genetic factors as well as environmental stress (Campisi et al., 2019), affects the brain along with the rest of the body, causing changes in cognitive functions (Sugiura, 2016; Gonzales et al., 2022). Cognitive aging is affected by changes in the structure of neurons as well as neural atrophy, occurring especially in the frontal and temporal regions of the brain (Fjell and Walhovd, 2010; Isaev et al., 2019). Impairments in cognitive performance are seen specifically in executive function, processing speed, memory, and reasoning (Salthouse, 2019). Cognitive decline is also closely interlinked with poor emotional wellbeing. For example, depression has been recognized as a significant risk factor for the development of dementia (Kivipelto et al., 2018) and is also a common symptom in early dementia (Kubo et al., 2019). Depression and psychosocial stress can cause changes at the epigenetic level, which can result in accelerated neurobiological aging (Boccardi and Boccardi, 2019; Palma-Gudiel et al., 2020; Polsky et al., 2022). Furthermore, the reduction of social networks and increased loneliness associated with aging can severely impair cognitive, emotional, and even physical wellbeing (Boss et al., 2015; O’Rourke et al., 2018; Valtorta et al., 2018). Thus, the interconnectedness of cognitive, emotional, physical, and social changes in later life makes it crucial to find holistic ways to promote wellbeing in older adults.

Despite these risks that aging poses for the wellbeing of individuals, the brain remains plastic throughout life and is able to adapt to changes induced by the aging process (Sugiura, 2016). For example, the brain can compensate for lost volume and functioning of specific areas by recruiting frontal areas more extensively and reducing bilateral asymmetry in carrying out functions (Reuter-Lorenz and Park, 2014). Furthermore, practice and training different skills can have positive effects on brain structure and function as well as cognition even in older adults (Reuter-Lorenz and Park, 2014; Cespón et al., 2018; Nguyen et al., 2019). There are also a number of protective factors related to lifestyle, such as education and different leisure activities, which have been found to preserve cognitive functioning in older age (Kivipelto et al., 2018; Song et al., 2022).

Among beneficial leisure activities throughout the lifespan music has been considered particularly promising and has received increasing interest among researchers during the last couple of decades. Music is a source of enjoyment, learning, and wellbeing in life and a particularly rich stimulus for the brain, which is backed by converging evidence from research across different fields (Särkämö and Sihvonen, 2018). Music engages the brain widely, involving a large-scale, bilateral network of cortical and subcortical regions that mediate the multitude of cognitive, motor, emotional, and social functions related to music processing (Alluri et al., 2012; Zatorre and Salimpoor, 2013). Musical training has been found to induce structural and functional neuroplastic changes along the auditory, motor, and somatosensory as well as multimodal integration pathways (Herholz and Zatorre, 2012) and enhance cognitive performance with transfer effects on executive function, attention, and memory (Benz et al., 2016). Notably, evidence suggests that musical practice induces structural brain plasticity in older adults in regions that are vulnerable to atrophy with age (Sluming et al., 2002; Chaddock-Heyman et al., 2021), and that music making can slow down normal age-related changes in brain structure (Rogenmoser et al., 2018). Training to play a musical instrument at old age and instrumental musical practice throughout life have been linked to better cognitive flexibility, processing speed, working memory, and verbal and non-verbal memory in older adults (Bugos et al., 2007; Hanna-Pladdy and Gajewski, 2012; Mansens et al., 2018; for a review, see Román-Caballero et al., 2018). Furthermore, music interventions have been shown to enhance cognition and mood even in mild cognitive impairment and dementia (Särkämö et al., 2014; Hofbauer et al., 2022; Ito et al., 2022; Jordan et al., 2022).

Although the neurocognitive impact of instrumental musical training has been extensively studied, we know relatively little about the effects of singing on neuroplasticity and cognitive functions during aging. Singing is a multi-domain process for the brain, requiring the complex interplay of auditory, vocal-motor, linguistic, cognitive, and emotional processes. Based on neuroimaging studies, singing entails the continuous interaction of two cortical systems, the parietal-frontal (dorsal) vocal production pathway and the temporal-frontal (ventral) auditory perception pathway, which work together as a loop to enable fine vocal motor control based on somatosensory and auditory feedback (Zarate, 2013; Kleber et al., 2017; Segado et al., 2021). Compared to speech production, singing production (with lyrics) engages portions of fronto-temporal brain regions more strongly and with more right lateralization (Callan et al., 2006; Ozdemir et al., 2006). In addition, brain structures linked to attention, working memory, rhythm, and emotion are engaged during singing, including prefrontal, limbic, and cerebellar structures (Alluri et al., 2013; Whitehead and Armony, 2018; Wang et al., 2019).

Choir singing is the most popular musical activity engaging 37 million singers in Europe, and participation especially in senior choirs is growing rapidly (European Choral Association, 2015). The singing-related brain processes together with the social interaction and goal-directed learning (learning to sing and perform polyphonic song arrangements) make choir singing a particularly promising tool for promoting cognitive reserve and psychological and social wellbeing in aging. Findings from previous research indicate that group singing can improve mental health and emotional and social wellbeing in adults with a mental health condition (Williams et al., 2018). The emotional benefits of singing have been linked to the secretion of endocannabinoids, immunoglobulins, and cortisol (Stone et al., 2018). In addition, singing has been connected to physiological benefits, such as improving cardiorespiratory functions (Bernardi et al., 2017; Fu et al., 2018), and it can even aid in rehabilitation of motor and cognitive functioning as well as speech production in aging-related neurological illnesses, such as stroke, dementia, and Parkinson’s disease (Harrison et al., 2017, 2019; Sihvonen et al., 2020). In older adults, regular participation in choir singing has been shown to reduce anxiety, depression, and loneliness; improve self-evaluated quality of life (QOL), physical health, and interest in life; and increase general activity (Johnson et al., 2013, 2020; Coulton et al., 2015). Furthermore, previous findings indicate that choir singing may benefit verbal fluency in older adults (Fu et al., 2018; Pentikäinen et al., 2021) as well as enhance the neural processing of auditory and speech stimuli (Dubinsky et al., 2019; Hennessy et al., 2021; Pentikäinen et al., 2022).

Previous studies on the effects of choir singing in older adults have been either cross-sectional studies or relatively short (3–6 months) intervention studies, and there are currently no longitudinal studies exploring the long-term effects of choir singing in the elderly over years. Here, we report the results of a two-year follow-up study where the cognitive, emotional, and social wellbeing of a cohort of elderly choir singers (*N* = 107) and demographically matched non-singer control subjects (*N* = 62) were followed using questionnaires and neuropsychological tests. We aimed to find out whether, firstly, choir singers would show less decline in cognitive performance as well as in self-reported cognitive functioning, mood, social wellbeing, and QOL compared to the controls over the two-year period. Secondly, we explored if the duration of the singing hobby (number of choir singing years) would be associated with the possible long-term benefits of choir singing, expecting longer singing experience to be linked with less decline.

## Materials and methods

### Participants and study design

The study was approved by the Ethical Review Board in the Humanities and Social and Behavioral Sciences in the University of Helsinki. We recruited 107 choir singers and 62 demographically matched non-singer control subjects from adult education centers, independent choirs, and local senior citizens’ associations in the capital region of Finland via presentations and advertisements. The inclusion criteria for participants were that they were 60 years or older, Finnish speaking, had no neurological or severe psychiatric disorders, no severe sleep disorders, no severe hearing loss, and were not taking medications affecting the central nervous system. Regarding choir singing, the inclusion criteria for choir singers was that they sang in a choir led by a trained conductor, practiced at least once a week, and performed with the choir at least twice a year. An additional criterion for the control subjects was that they had not been singing in a choir during the past 10 years. Demographic information of the participants is specified in Table 1. Written informed consent was obtained from all participants. Participants were compensated for their time with vouchers.

**TABLE 1:** Demographic characteristics of the participants.

		Time	*N*	Gender (Female/ Male)	Age	Education level (ISCED)	Living situation (alone/ Together)	Choir singing in years
Questionnaires	Choir	1	107	77/30	70.8 (5.4)	4.9 (1.7)	36/66	15.8 (14.3)
		2	104	75/29	72.0 (5.4)	4.9 (1.7)	40/62	16.1 (14.5)
		3	101	73/28	73.0 (5.4)	4.9 (1.7)	40/60	16.3 (14.5)
	Control	1	62	53/10	70.6 (6.6)	4.9 (2.0)	31/30	–
		2	58	48/10	71.3 (6.1)	4.9 (2.0)	29/29	–
		3	50	42/8	72.1 (6.3)	5.1 (1.8)	23/27	–
Neuropsychological tests	Choir	1	45	29/16	71.3 (5.7)	4.8 (1.5)	16/28	17.5 (16.0)
		2	38	25/13	72.1 (6.0)	4.8 (1.6)	15/23	17.4 (16.1)
		3	33	23/10	72.4 (6.1)	4.7 (1.5)	14/19	18.4 (16.9)
	Control	1	41	33/8	70.5 (6.7)	4.7 (2.1)	20/20	–
		2	35	28/7	70.8 (5.9)	4.8 (2.1)	16/19	–
		3	27	22/5	71.9 (6.3)	5.2 (1.9)	10/17	–

Data are mean (SD) unless otherwise reported. ISCED, international standard classification of education (range 1 = primary education, 2 = lower secondary, 3 = upper secondary, 4 = post-secondary non-tertiary, 5 = short cycle tertiary, 6 = bachelor or equivalent, 7 = master or equivalent, 8 = doctoral level).

We conducted a follow-up study over 3 years from 2017 to 2020. Due to the COVID-19 pandemic and the closing of choir activities and the detrimental psychosocial effects (e.g., mobility restrictions) caused by it, the data collected within the final year (2020) of the follow-up could not be used in this study. Hence, here we address the first 2 years (from 2017 to 2019) of the follow-up. While the majority of participants were recruited in 2017, a small set of participants joined the study in 2018 (*N* = 8), and a one-year follow-up from them was included in the analysis. All participants filled out questionnaires sent to their homes once a year, and a subgroup of participants (*N* = 86, 45 choir singers and 41 controls) also took part in neuropsychological tests each year. Altogether 12 participants dropped out from the study during the two-year follow-up due to health or other personal reasons. Thus, the final sample at different time points was 169 participants (107 choir singers and 62 controls) at baseline, 162 participants (104 choir singers and 58 controls) at the one-year follow-up, and 151 participants (101 choir singers and 50 controls) at the two-year follow-up.

### Questionnaires

The questionnaires used in this study were designed to measure participants’ subjective cognitive functioning, emotional and social wellbeing, and QOL. Detailed descriptions of each questionnaire are listed in Table 2. The cognitive failures questionnaire (CFQ; Broadbent et al., 1982) and the prospective and retrospective memory questionnaire (PRMQ; Smith et al., 2000) were used to assess cognitive functioning. Emotional wellbeing was assessed with the Center for Epidemiological Studies Depression scale (CES-D; Radloff, 1977), which is designed to screen symptoms of clinical depression. Social wellbeing was measured using the social provisions scale (SPS; Cutrona and Russell, 1987), a questionnaire asking about the level of support, attachment, and integration experienced by the participants in their social relationships as well as their experiences of being valued by others. The SPS has six subscales: the attachment scale measures the experiences of having close and warm relationships; the integration subscale assesses feelings of belonging to a group and knowing people with similar values; the reassurance of worth subscale includes questions about feeling valued; the reliable alliance scale evaluates the experiences of getting help in relationships when needed; the guidance subscale evaluates the possibilities to share thoughts and worries with someone and get advice; and the opportunity of nurturance subscale assesses the feelings of being needed and being able to offer help and advice to someone else. World Health Organization’s quality of life questionnaire (WHOQOL-Bref; The WHOQOL Group, 1998) was used to measure the physical, psychological, social, and environmental aspects of QOL experienced by the participants. The WHOQOL-Bref questionnaire divides QOL assessment to four different categories: the physical subscale covers questions about physical health and ability to maintain activities of daily living; the psychological subscale measures satisfaction with oneself and experiences of meaning in life as well as feelings of depression, anxiety or despair; the social subscale includes assessment of social relationships; and the environmental subscale measures feelings of safety in the living environment, accessibility of services, and the possibilities to influence and take part in everyday activities.

**TABLE 2:** Description of used questionnaires and neuropsychological tests.

Questionnaires	Measure	Description
Cognitive function	CFQ	25 items measuring cognitive failures in different everyday situations involving perception, attention, memory and motor functions
	PRMQ	16 items measuring prospective and retrospective memory
Depression	CES-D	20 items measuring depressive symptoms
Social wellbeing	SPS	24 items measuring level of support from social relationships 6 scales: attachment, social integration, reassurance of worth, reliable alliance, guidance, and opportunity of nurturance
QOL	WHOQOL-Bref	26 items measuring different aspects of quality of life 4 scales: physical, psychological, social, and environmental QOL 2 separate items: overall QOL and general health
**Neuropsychological tests**		
General cognition	MoCA	6 short tasks measuring visuospatial functions, verbal abilities, memory, attention and orientation
EF: verbal flexibility	Phonemic fluency Semantic fluency	List verbally as many words as possible during 60 s starting with the letter S List verbally as many animals as possible during 60 s
EF: shifting	FAT	Trail Making Test: connect numbers (Part A) and numbers and letters (Part B) as fast as possible
EF: inhibition	Simon task	Respond to red/blue square appearing on the left/right side of screen by pressing a button (congruent and incongruent trials)
Processing speed	WAIS-IV	Visual search: copy symbols corresponding to numbers (2 min) Coding: search rows of symbols for target symbols (2 min)
Working memory	WAIS-IV	Digit span: recall lists of numbers in different order Arithmetic: solve verbally presented arithmetic tasks
	FAT	Visual span: recall visuospatial patterns
EM: immediate	WMS-III	Logical memory I: recall a story immediately after hearing it Word lists I: recall a list of words immediately after hearing it
EM: delayed	WMS-III	Logical memory II: recall a story after 30 min delay Word lists II: recall a list of words after 30 min delay
Vocabulary	WAIS-IV	Vocabulary: explain the meaning of words

CFQ, cognitive failures questionnaire; PRMQ, prospective and retrospective memory questionnaire; CES-D, Center for Epidemiologic Studies Depression scale; SPS, social provisions scale; WHOQOL-Bref, quality of life questionnaire of the World Health Organization; MoCA, Montreal Cognitive Assessment; EF, executive function; FAT, flexible attention test; WAIS-IV, Wechsler Adult Intelligence Scale IV; EM, episodic memory; WMS-III, Wechsler Memory Scale III.

### Neuropsychological tests

Neuropsychological tests were conducted by licensed psychologists or psychology Master’s students (supervised by a psychologist) in a quiet testing room at the laboratory of the Cognitive Brain Research Unit, University of Helsinki. The psychologists agreed on testing practices beforehand, met regularly, and consulted with each other in order to maintain similar practices. The testing took 1.5 h and was administered at the same time of day at each time point for each participant. Detailed list of the neuropsychological test battery is presented in Table 2. The battery covered seven cognitive domains: global cognition, executive functions, processing speed, working memory, episodic memory, and verbal cognition.

We used the Montreal Cognitive Assessment (MoCA; Nasreddine et al., 2005) to evaluate global cognition. Executive functions were divided to three subdomains: (i) verbal flexibility was assessed with the phonemic and semantic fluency tests (Lezak et al., 2012); (ii) shifting was assessed with a computerized (tablet) modification of the Trail Making Test included in the flexible attention test (FAT) developed at the Finnish Institute of Occupational Health (Järnefelt et al., 2018), and (iii) inhibition was assessed with the Simon task (Simon and Rudell, 1967; Martin et al., 2012) using the Presentation software (Neurobehavioral Systems, Inc., Berkeley, CA, USA). Symbol search and Coding subtests of the Wechsler Adult Intelligence Scale IV (WAIS-IV; Wechsler, 2008) were used to evaluate processing speed, and sum of the raw scores was used in the analysis. Working memory was assessed with the digit span and Arithmetic subtests of WAIS-IV and a tablet version of the Corsi Block-tapping test included in the FAT (Kessels et al., 2000), and the sum score of these three tests was used as the working memory variable. Episodic memory was evaluated using the Logical memory and Word lists subtests of the Wechsler Memory Scale III (WMS-III; Wechsler, 1997), which were also combined for the analysis. Verbal cognition was evaluated with the Vocabulary subtest from WAIS-IV. For the memory tests (one subtest of MoCA, Logical memory, Word lists), we used two alternative word lists (formulated to resemble the features of the original word list as closely as possible) and one alternative story (from the Finnish KAT test; Manninen et al., 2015) in addition to the originals in order to avoid learning effects. The order of presentation of the alternative test versions was randomized among participants.

### Statistical analysis

To assess change over time in the questionnaire and test-based measures, we conducted a linear mixed model (LMM) analysis for raw scores of each variable. For the questionnaire data, we used Group (choir/control), Time (T1/T2/T3), Gender (female/male), and Living mode (alone/not alone) as fixed variables and Age (in years) as a covariate. For the neuropsychological test data, the fixed variables in the model were Group, Time, Education level (international standard classification of education, range 1 = primary education to 8 = doctoral level), and Gender; Age was used as a covariate. The interaction between Group and Time was included in the models. The background variables used in the model were based on the differences observed between groups at baseline reported in our previous article from the same sample (Pentikäinen et al., 2021). *Post hoc*, we calculated pairwise comparisons on the estimated marginal means to assess changes in the scores within groups. Bonferroni correction was used in *post hoc* pairwise tests to adjust for multiple comparisons.

Finally, we assessed whether there was a correlation between the duration of the choir hobby (number of choir singing years) as well as the frequency of singing during the study period (assessed on a 6-point Likert scale at each time point, from 0 = never to 5 = daily) and questionnaire or neuropsychological test scores at T1 as well as their change from T1 to T3 in those variables where significant group differences were observed in the LMM analyses. Partial correlation was calculated with the background variables included as covariates. This way, we sought to assess how choir singing experience may mediate the long-term change in the questionnaire and neuropsychological test variables. The data were analyzed using SPSS version 28 (IBM SPSS Statistics, 2021).

## Results

### Long-term effects of choir singing on subjective cognitive, emotional, and social wellbeing: questionnaire results

**FIGURE 1:**
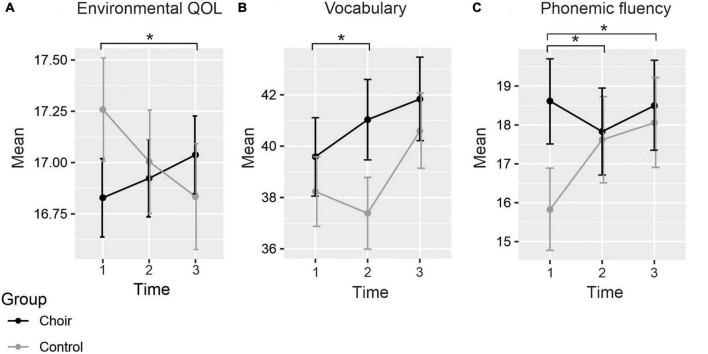
Mean scores of **(A)** environmental QOL, **(B)** vocabulary, and **(C)** phonemic fluency at each time point. Significant differences between groups in the change of the scores marked with asterisks. Standard errors marked with vertical lines.

Mean scores of the questionnaires assessing subjective cognitive functioning (CFQ, PRMQ), depression (CES-D), social wellbeing (SPS), and QOL (WHOQOL-Bref) in both Groups at each Time point are presented in Table 3. The LMM produced a significant Group × Time interaction in the environmental subscale of WHOQOL-Bref [F(2, 295.5) = 3.34, *p* = 0.037], by which the change in scores from T1 to T3 was significantly different between groups [t(298.3) = 2.57, *p* = 0.011] (see Figure 1A), showing increase in choir singers and decrease in non-singers. However, the change was not significant within either group separately (choir: *p* = 0.369; control: *p* = 0.159). No significant Group × Time interactions were observed in any of the other questionnaire measures. Significant main effects of Time were for found for overall QOL [F(2, 322.3) = 4.49, *p* = 0.012], CFQ [F(2, 343.8) = 25.9, *p* < 0.001], and the reassurance of worth subscale of the SPS [F(2, 322.7) = 6.00, *p* = 0.003]. These main effects indicated that over groups, overall QOL decreased over time (T1–T2: *p* = 0.723, T1–T3: *p* = 0.040), whereas increase was seen in self-evaluated cognitive failures (T1–T2: *p* < 0.001, T1–T3: *p* < 0.001) as well as feelings of being valued by others (T1–T2: *p* = 0.019, T1–T3: *p* = 0.061).

**TABLE 3:** Mean scores (SE) of questionnaires in both groups at each time point.

Questionnaire measure	T	Choir singers (*N* = 107)	Controls (*N* = 62)	Group *p*-value F(df)	Time *p*-value F(df)	G × T *p*-value F(df)
WHOQOL-Bref: environmental QOL	1	16.8 (0.2)	17.3 (0.3)	0.592	0.794	**0.027**
	2	16.9 (0.2)	17.0 (0.2)	0.29 (1,159.3)	0.23 (2,334.6)	3.34 (2,295.5)
	3	17.0 (0.3)	16.9 (0.3)			
WHOQOL-Bref: physical QOL	1	15.8 (0.2)	15.6 (0.3)	0.368	0.458	0.289
	2	15.7 (0.2)	15.8 (0.3)	0.82 (1,163.6)	0.78 (2,341.7)	0.90 (2,299.2)
	3	15.8 (0.2)	15.5 (0.3)			
WHOQOL-Bref: psychological QOL	1	15.8 (0.2)	15.6 (0.3)	0.514	0.780	0.289
	2	15.7 (0.2)	15.8 (0.3)	0.43 (1,160.6)	0.25 (2,341.1)	1.48 (2,298.1)
	3	15.8 (0.2)	15.5 (0.3)			
WHOQOL-Bref: social QOL	1	15.8 (0.3)	15.4 (0.4)	0.108	0.041	0.176
	2	15.4 (0.3)	15.0 (0.4)	2.62 (1,161.5)	3.23 (2,340.7)	1.72 (2,296.2)
	3	15.8 (0.3)	14.8 (0.4)			
WHOQOL-Bref: overall QOL	1	4.2 (0.1)	4.3 (0.1)	0.636	**0.012**	0.359
	2	4.1 (0.1)	4.2 (0.1)	0.22 (1,162.7)	4.49 (2,322.3)	0.75 (2,303.2)
	3	4.1 (0.1)	4.1 (0.1)			
WHOQOL-Bref: general health	1	4.0 (0.1)	3.9 (0.1)	0.158	0.401	0.288
	2	4.0 (0.1)	3.8 (0.1)	2.01 (1,163.7)	0.92 (2,325.0)	1.30 (2,301.4)
	3	4.0 (0.1)	3.7 (0.1)			
CES-D total score	1	9.7 (0.7)	10.1 (1.0)	0.341	0.125	0.204
	2	10.3 (0.7)	10.9 (1.0)	0.91 (1,164.1)	2.09 (2,337.4)	1.60 (2,301.6)
	3	9.8 (0.7)	11.7 (1.0)			
CFQ total score	1	23.2 (1.3)	23.0 (1.7)	0.826	**< 0.001**	0.613
	2	28.1 (1.3)	27.0 (1.7)	0.05 (1,162.4)	25.9 (2,343.8)	0.49 (2,299.9)
	3	27.7 (1.3)	27.9 (1.7)			
PRMQ total score	1	31.3 (0.9)	31.6 (1.2)	0.952	0.089	0.074
	2	32.6 (0.9)	31.3 (1.2)	0.004 (1,164.2)	2.44 (2,343.7)	2.62 (2,304.7)
	3	32.3 (0.9)	33.0 (1.2)			
SPS total score	1	84.2 (0.8)	82.6 (1.1)	0.244	0.540	0.931
	2	84.5 (0.8)	83.1 (1.1)	1.37 (1,160.0)	0.62 (2,332.3)	0.07 (2,295.8)
	3	84.5 (0.9)	83.3 (1.2)			
SPS: attachment	1	14.5 (0.2)	13.9 (0.3)	0.107	0.236	0.398
	2	14.2 (0.2)	13.8 (0.3)	2.63 (1,156.8)	1.45 (2,322.0)	0.92 (2,294.4)
	3	14.1 (0.2)	13.9 (0.3)			
SPS: social integration	1	14.1 (0.2)	13.6 (0.2)	0.064	0.159	0.596
	2	14.2 (0.2)	14.0 (0.2)	3.47 (1,163.1)	1.85 (2,323.9)	0.52 (2,305.3)
	3	14.3 (0.2)	13.8 (0.2)			
SPS: reassurance of worth	1	13.0 (0.2)	13.0 (0.2)	0.869	**0.003**	0.901
	2	13.4 (0.2)	13.4 (0.2)	0.03 (1,160.6)	6.00 (2,322.7)	0.10 (2,299.8)
	3	13.4 (0.2)	13.4 (0.3)			
SPS: reliable alliance	1	14.7 (0.2)	14.6 (0.2)	0.813	0.146	0.975
	2	14.9 (0.2)	14.8 (0.2)	0.06 (1,158.7)	1.94 (2,320.9)	0.03 (2,300.3)
	3	15.0 (0.2)	15.0 (0.2)			
SPS: guidance	1	14.8 (0.2)	14.3 (0.2)	0.177	0.585	0.413
	2	14.5 (0.2)	14.4 (0.3)	1.84 (1,157.0)	0.54 (2,322.9)	0.89 (2,295.7)
	3	14.7 (0.2)	14.3 (0.3)			
SPS: opportunity of nurturance	1	13.0 (0.2)	13.2 (0.3)	0.937	0.954	0.457
	2	13.2 (0.2)	13.0 (0.3)	0.01 (1,162.0)	0.05 (2,324.5)	0.79 (2,302.1)
	3	13.0 (0.2)	13.1 (0.3)			

Significant Group × Time interactions are shown in bold font. SE, standard error; T, time; G, Group; WHOQOL-Bref, quality of life questionnaire of the World Health Organization; CES-D, Center for Epidemiologic Studies Depression scale; CFQ, cognitive failures questionnaire; PRMQ, prospective and retrospective memory questionnaire; SPS, social provisions scale.

### Long-term effects of choir singing on cognitive functioning: neuropsychological test results

Mean raw scores of the neuropsychological tests assessing the six cognitive domains (general cognition, executive function, processing speed, working memory, episodic memory, and verbal cognition) in both Groups at each Time point are presented in Table 4. There was a significant Group × Time interaction in the vocabulary score [F(2, 115.1) = 3.45, *p* = 0.035], whereby the difference between the groups significantly increased from T1 to T2 [t(115.3) = 2.31, *p* = 0.023] (see Figure 1B). Although the direction of the change differed between groups, the change from T1 to T2 was not statistically significant within choir singers (*p* = 0.132) or controls (*p* = 0.705). Main effect of Time was significant [F(2,133.7) = 9.08, *p* < 0.001], showing an increase between T1 and T3.

**TABLE 4:** Mean scores (SE) of neuropsychological tests in both groups at each time point.

Cognitive domain	T	Choir singers (*N* = 107)	Control subjects (*N* = 62)	Group *p*-value F(df)	Time *p*-value F(df)	G × T *p*-value F(df)
Vocabulary	1	39.6 (1.5)	38.2 (1.4)	0.311	**0.002**	**0.036**
	2	41.0 (1.6)	37.4 (1.4)	1.04 (1,67.4)	6.85 (2,114.8)	3.43 (2,114.6)
	3	41.8 (1.6)	40.5 (1.5)			
Executive function: verbal flexibility Phonemic fluency	1	18.4 (1.1)	15.7 (1.0)	0.330	0.280	**0.033**
	2	17.6 (1.1)	17.5 (1.1)	0.96 (1,71.9)	1.29 (2,131.0)	3.50 (2,130.7)
	3	18.2 (1.1)	17.8 (1.1)			
Semantic fluency	1	24.1 (1.3)	22.6 (1.2)	0.273	0.966	0.701
	2	24.7 (1.3)	23.0 (1.3)	1.22 (1,75.1)	0.03 (2,131.8)	0.37 (2,131.6)
	3	25.3 (1.4)	22.8 (1.3)			
Executive function: Shifting	1	35.7 (5.2)	31.8 (5.1)	0.318	0.875	0.991
	2	33.5 (5.5)	29.6 (5.7)	1.01 (1,69.3)	0.13 (2,128.3)	0.02 (2,127.8)
	3	34.4 (5.8)	32.0 (6.1)			
Executive function: Inhibition	1	103.2 (8.0)	92.1 (7.8)	0.354	0.669	0.678
	2	93.4 (8.2)	89.8 (8.4)	0.87 (1,74.2)	0.40 (2,125.8)	0.41 (2,125.1)
	3	94.0 (9.2)	92.6 (9.3)			
Global cognition	1	26.1 (0.6)	25.4 (0.5)	0.844	0.425	0.092
	2	25.5 (0.6)	25.8 (0.6)	0.04 (1,75.8)	0.86 (2,133.6)	2.44 (2,133.4)
	3	25.7 (0.6)	25.8 (0.6)			
Processing speed	1	72.2 (2.7)	72.8 (2.7)	0.337	0.221	0.991
	2	74.9 (2.9)	76.4 (2.8)	0.93 (1,76.1)	1.53 (2,131.1)	0.004 (2,130.9)
	3	75.6 (3.1)	77.6 (3.0)			
Working memory	1	40.8 (1.3)	39.1 (1.2)	0.257	0.090	0.556
	2	40.4 (1.3)	39.8 (1.2)	1.31 (1,71.3)	2.46 (2,120.1)	0.90 (2,120.0)
	3	42.0 (1.3)	40.2 (1.3)			
Episodic memory: Immediate recall	1	41.6 (1.4)	40.6 (1.3)	0.265	0.534	0.356
	2	43.5 (1.4)	40.5 (1.4)	1.26 (1,74.6)	0.63 (2,132.9)	1.03 (2,132.6)
	3	42.8 (1.5)	40.0 (1.5)			
Episodic memory: Delayed recall	1	16.2 (1.2)	15.2 (1.1)	0.636	0.219	0.918
	2	17.4 (1.2)	16.6 (1.2)	0.23 (1,72.4)	1.54 (2,129.5)	0.11 (2,129.3)
	3	17.3 (1.3)	15.9 (1.3)			

Significant Group × Time interactions are shown in bold font. SE, standard error; T, time; G, Group.

A significant Group × Time interaction was also found for the phonemic fluency score [F(2,130.7) = 3.51, *p* = 0.033]. This was explained by a significant difference in how the scores between groups changed from T1 to T2 [t(130.9) = −2.42, *p* = 0.017] and from T1 to T3 [t(132.0) = −2.03, *p* = 0.044; see Figure 1C], with different trajectories in scores between the groups from baseline to each follow-up measurement. *Post hoc* analysis revealed that at baseline scores were significantly higher in choir singers (*p* = 0.023), but their scores did not change significantly over time (*p*-values > 0.85). The performance in controls significantly improved from T1 to T3 (*p* = 0.031), and marginally from T1 to T2 (*p* = 0.068). No significant main effects or Group × Time interactions were observed in any of the other neuropsychological tests.

### Association between choir singing experience and long-term benefits: correlation results

**FIGURE 2:**
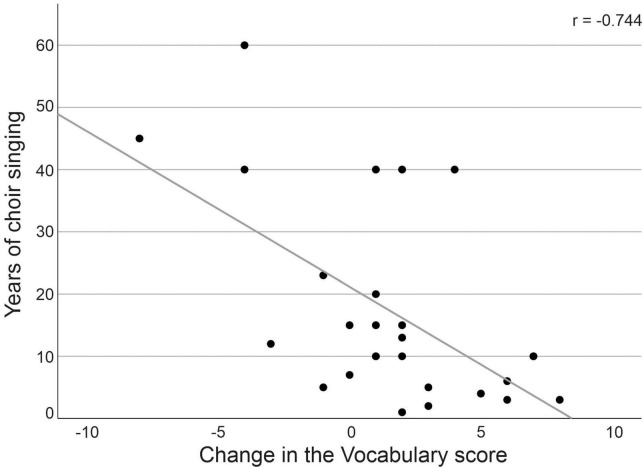
Correlation between number of choir singing years and vocabulary score change from T1 to T3.

Finally, we examined if the number of choir singing years or frequency of singing during the study period were associated with the scores of environmental QOL, vocabulary and phonemic fluency at T1. We found no significant correlations between either the years of singing or singing frequency and any of the measures (*p* > 0.187). We further explored if choir singing years or frequency were connected with the environmental QOL and vocabulary scores, in which an increasing trend was observed within choir singers. We found a significant negative correlation between the number of choir singing years and the change in the vocabulary performance between T1 and T3 [r(19) = −0.744; *p* = 0.002; Figure 2] within the choir singers, indicating that those with shorter singing experience demonstrated greater increase in the vocabulary score. The years of singing did not correlate with the environmental QOL (*p* = 0.293). No correlations were found between the frequency of singing during the study period and with the change in the vocabulary score (*p* = 0.314) or the environmental QOL (*p* = 0.832).

## Discussion

In this study, our aim was to determine whether regular choir singing can have long-term benefits on cognitive, emotional, and social functioning in healthy older adults. Previous studies in older adults have identified benefits of group singing on various aspects of wellbeing, including cognition, physical health, mood, social wellbeing, and QOL (Johnson et al., 2013, 2020; Coulton et al., 2015; Fu et al., 2018; Pentikäinen et al., 2021, 2022). Critically, however, evidence from longer-term follow-up periods is still lacking. We followed up a sample of choir singers and non-singing control subjects of 60 years and above for 2 years including three annual measurement points with subjective measures of cognitive and emotional functioning, social wellbeing and QOL as well as neuropsychological tests of global cognition, executive functions, episodic memory, and verbal cognition.

We expected that the choir singers would show less decline in cognitive performance as well as in self-reported cognitive functioning, mood, social wellbeing, and QOL compared to the controls. Our results provided some limited support for these hypotheses. No clear benefits of choir singing on QOL were observed; the groups differed only in the direction of self-reported scores in social wellbeing in the environmental domain of QOL, but the change over time was not significant within either group nor was there a difference between groups at T1. In the cognitive domain, choir singers demonstrated an increasing trend in their performance in the vocabulary task over the first year compared to the controls with a declining trend. However, over the 2 years of follow-up, both groups established a significant improvement in the task. In contrast, while the choir singers initially showed higher phonemic fluency, the controls improved in this task over time to the level of the choir singers. These results are discussed in more detail below.

In healthy older adults, QOL has been reported to generally decline with age, and changes in QOL over time are known to be linked to changes in mood (e.g., depression), functional abilities (e.g., activities of daily living), financial situation, and social relationships (e.g., loneliness) (Webb et al., 2011; Brett et al., 2019). Similarly, we observed a significant decline across groups in QOL, as measured by the overall QOL item of the WHOQOL-Bref questionnaire, over the two-year period. In previous studies assessing choir singing in healthy older adults, choir singers have been reported to show better QOL compared to controls (Johnson et al., 2017; Pentikäinen et al., 2021), and choir singing interventions have been shown to improve QOL (Coulton et al., 2015) as well as reduce loneliness and increase interest in life (Johnson et al., 2020) over a 6-month follow-up in randomized controlled trials. In our study, the only QOL domain, in which there was a different longitudinal trajectory between groups was the environmental subscale of WHOQOL-Bref where choir singers showed enhancement compared to the decline observed in non-singers. However, as there were no significant changes within either group, we cannot conclude that choir singing would have benefitted this aspect of QOL over the two-year period. In our previous study, we found that choir singers experienced greater satisfaction with their general health compared to controls as well as greater social integration (Pentikäinen et al., 2021). These factors could be reflected also in the environmental QOL, which measures the experiences of being able to participate in and influence activities and services in their living environment as well as the feelings of safety. This would also fit the general view of singing and social music making as an activity that is linked to the experience of pleasure and reward, that can support positive mood and social participation, and that enable social participation and connectedness with others (Noice et al., 2014; Greenberg et al., 2021). However, our results seem to indicate that the effects of choir singing on QOL might only be observed in the short-term. More research is needed to define whether choir singing can benefit QOL over longer periods of time. Follow-up periods over several years may also be better able to provide answers on the longitudinal effects compared to the two-year follow-up in our study. Furthermore, as the control group of the study was recruited from other hobby groups in the adult education centers, it could be that the controls were also motivated to learn new skills and maintain an active lifestyle, which can promote their QOL.

Overall, subjects reported subjective increase in everyday cognitive mistakes during the study period, which was not seen in test-based measures. Subjective cognitive decline is common among older adults, and is not always related to objective cognitive impairment (Jessen et al., 2020). It is possible that by participating in the study regularly, the participants started to observe their cognitive functions more and as a result, paid attention to common everyday cognitive failures more than previously. On the other hand, training effects on the neuropsychological tests administered several times during the follow-up can also possibly obscure some of the decline. There were no differences between groups, indicating that choir singing did not protect against subjective cognitive decline.

In the neuropsychological tests, no clear benefits over time were observed in the choir singers compared to controls. Differences between groups over the two-year follow-up were seen only in tasks measuring verbal cognition and flexibility. This was manifested by performance in the Vocabulary subtest of WAIS-IV, which requires active retrieval of word knowledge and essentially activation of lexico-semantic networks in the brain, and in the phonemic fluency task, which taps the executive demands of fast retrieval and production of lexicon constrained by a phonological rule. In the vocabulary task, performance of both groups enhanced from T1 to T3, but the change differed between groups only from T1 to T2, where choir singers’ score increased while the controls’ decreased; however, this change was not significant within either group. Thus, the choir singers showed a steadier increase in the vocabulary score compared to controls, but due to the lack of significant change within either group from T1 to T2 the cause of this difference between groups remains unclear. The scores of the choir singers were overall slightly higher, although not reaching statistical significance. A previous finding showed a link between social leisure activities and slower decline in vocabulary performance over a five-year period (Sanz Simon et al., 2022). Furthermore, studies in healthy old age have shown no change or improvement in the vocabulary task over follow-up periods of 3 and 6 years (Ghisletta and Lindenberger, 2004; Royall et al., 2005, respectively). These studies suggested that unlike in many other cognitive tasks in old age, the lack of decline in vocabulary was due to task-specific learning. Our results support these findings, indicating that learning occurred in both groups in the two-year follow-up.

In the phonemic fluency task, the choir singers had significantly higher score already at baseline [as reported previously in Pentikäinen et al. (2021)] and they remained stable across time whereas the scores of the controls were initially lower but increased over time. A plausible interpretation of the result could be that choir singers demonstrated scores at ceiling in this age group already at baseline, allowing no significant further improvements. The increase in the scores of controls probably demonstrates a learning effect in the task. No difference between the groups was found in the semantic fluency task, however. Generally, the phonemic task is considered more difficult, possibly enabling more variance in healthy samples. While these results need to be interpreted with some caution due to the relatively small sample size, possible test-retest learning effects in the tasks and the lack of connection between years of singing experience and phonemic fluency performance at T1, they indicate that choir singing may potentially be linked to benefits in rapid verbal execution in healthy older adults. This is supported by previous studies in older adults reporting that group-based musical activities (singing, instrument playing) are associated with improved verbal fluency (Hanna-Pladdy and Gajewski, 2012; Fauvel et al., 2014; Fu et al., 2018). Specifically, our results align with findings from Hanna-Pladdy and Gajewski (2012), who showed that older adult musicians performed significantly better in phonemic fluency than non-musicians, but not in semantic fluency. Fu et al. (2018) also reported a stronger improvement in phonemic than semantic fluency over a 12-week group-singing program in old adults.

Generally, word finding tends to become gradually slower and less accurate in older age (Verhaegen and Poncelet, 2013), which is linked to age-related decline in the language and cognitive control networks of the brain (Shafto and Tyler, 2014). There is evidence indicating that better functional connectivity in both language and cognitive networks could enable more preserved phonemic fluency performance at older age (Mohanty et al., 2021; Pistono et al., 2021). Striatal dopamine function has also been linked to verbal functions in aging (Berry et al., 2018; Li et al., 2020), and as musical activities and the pleasure they induce are associated with striatal dopaminergic activity (Zatorre and Salimpoor, 2013), singing practice can influence these functions in older adults. Moreover, singing production engages many of the frontotemporal brain regions and their connecting white matter pathways that are also involved in speech production and active memory retrieval of verbal information, but in a more bilateral or right-lateralized fashion (Callan et al., 2006; Ozdemir et al., 2006; Zarate, 2013; Kleber et al., 2017; Segado et al., 2021), which may underlie also the more general benefits of singing on speech production in aging. Neuroimaging studies on singing production in older adults are needed to establish the roles of the specific neural structures in mediating the positive effects of choir singing in the language network.

Interestingly, we also observed a negative correlation between choir singing years and change in the vocabulary score over the two-year follow-up, indicating that the improvement in the task was higher in those seniors who had started the choir singing hobby more recently (e.g., upon their retirement from working life). However, as the change in the score did not differ between groups from T1 to T3, it cannot be unequivocally concluded that choir singing started at older age benefits vocabulary performance. Furthermore, years of choir singing or the frequency of singing during the follow-up did not correlate with the score at T1 or its change from T1 to T3. Generally, better vocabulary performance in old age has been linked to greater engagement in leisure activities closely after retirement (Ihle et al., 2016). Vocabulary performance has been viewed as an index of cognitive reserve (Nogueira et al., 2022), reflecting the reduced susceptibility to age-related pathological brain changes, such as those in Alzheimer’s disease (Stern, 2012). Taken together, cognitively, physically, and socially stimulating leisure activities such as choir singing can possibly be a useful tool for supporting cognitive reserve in aging, but this hypothesis still requires more empirical support from studies with larger sample sizes and longer longitudinal follow-up.

The present study has some limitations. First, a small sample size limits the conclusions that can be drawn from the results and may, on the other hand, prevent the detection of subtle differences between groups. The sample sizes are also different for the questionnaire and neuropsychological measures, making it more complex to compare them and form a sense of overall wellbeing of the participants. Furthermore, while we corrected for multiple comparisons in the *post-hoc* testing, we did not use an omnibus Bonferroni correction across all outcome measures, which limits the conclusions that can be drawn from the results, and more research is needed to verify them. Another limitation concerns the follow-up period, which was relatively short and may not be sufficiently long to observe aging-related change in cognitive functions in healthy older adults. Thus, longer follow-up periods are needed to more comprehensively assess the possible cognitive benefits of choir singing in older adults. In addition, the repeated testing may in part prevent the detection of cognitive decline, as the participants gain experience of the tests and the testing process. This has been observed as a factor contributing to the smaller age-related decline observed in longitudinal compared to cross-sectional studies (Salthouse, 2019). Finally, we did not gather data about the previous choir singing experience of the control subjects, which, if it existed, may have contributed to the willingness of the controls to participate in the study and affected the results.

In conclusion, choir singing may induce enhanced and sustained performance in verbal fluency in older adults, although more evidence is needed to validate this result and examine the possible underlying mechanisms. Even though we did not find clear longitudinal group differences in neuropsychological measures, the difference between groups in phonemic fluency at T1 and the negative correlation between choir singing years and the change in the vocabulary score may point toward some possible benefits of choir practice at older age on cognitive functions. Long-term effects of choir singing on QOL remain unclear and require more research. Promoting the QOL and cognition of older adults is increasingly important as population ages. Choir singing is a low-cost and easy-to-arrange activity and can be applied in different settings reaching larger groups of individuals. Thus, it could provide an effective way to promote older adults’ QOL and wellbeing. However, more longitudinal research is needed to explore the possible connections between choir singing activity and healthy aging.

## Data availability statement

The raw data supporting the conclusions of this article will be made available by the authors, without undue reservation.

## Ethics statement

The studies involving human participants were reviewed and approved by the Ethical Review Board in the Humanities and Social and Behavioral Sciences in the University of Helsinki. The patients/participants provided their written informed consent to participate in this study.

## Author contributions

TS, EP, and AP contributed to conception and design of the study. EP, AP, and OL collected the data. EP and OL organized the database. EP performed the statistical analysis and wrote the first draft of the manuscript. TS and LK supervised the project. All authors contributed to manuscript revision, read, and approved the submitted version.
